# 2,2-Dimethyl-2,3-dihydro­pyrano[2,3-*a*]carbazol-4(11*H*)-one

**DOI:** 10.1107/S1600536808033849

**Published:** 2008-10-22

**Authors:** Makuteswaran Sridharan, Karnam J. Rajendra Prasad, Aimable Ngendahimana, Matthias Zeller

**Affiliations:** a Department of Chemistry, Bharathiar University, Coimbatore 641 046, Tamil Nadu, India; b Department of Chemistry, Youngstown State University, One University Plaza, Youngstown, OH 44555, USA

## Abstract

The title compound, C_17_H_15_NO_2_, was prepared from 1-hydroxy­carbazole and 3,3-dimethyl­acrylic acid with a mixture of AlCl_3_ and POCl_3_ as the cyclization catalyst. Owing to the presence of the –CMe_2_– group, the mol­ecule is not quite planar. In the crystal structre, strong N—H⋯O hydrogen bonds and weaker C—H⋯π inter­actions occur, and a slipped π–π stacking inter­action [centroid–centroid separation = 3.8425 (8) Å] is also observed.

## Related literature

Knölker & Reddy (2002 ▶) report on the isolation of pyran­ocarbazoles from various plant species, and Shanaza­rov *et al.* (1989 ▶) on their potential beneficial properties. Kavitha & Prasad (2003 ▶) describe the synthesis of compounds related to the title compound. Sridharan, Rajendra Prasad & Zeller (2008 ▶) report the structure of the 9-methyl derivative of the title compound. Sridharan, Rajendra Prasad, Ngendahimana & Zeller (2008 ▶) report the structure of the 10-methyl derivative of the title compound.
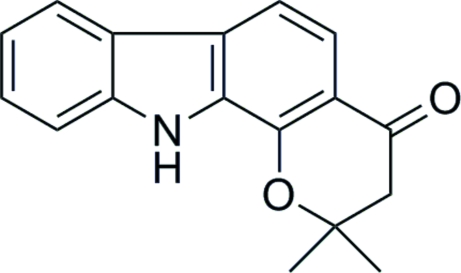


## Experimental

### 

#### Crystal data

C_17_H_15_NO_2_
*M* *_r_* = 265.30
Monoclinic, 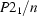

*a* = 5.9926 (5) Å
*b* = 14.3368 (12) Å
*c* = 15.6839 (13) Å
β = 95.270 (1)°
*V* = 1341.78 (19) Å^3^
*Z* = 4
Mo *K*α radiation
μ = 0.09 mm^−1^
*T* = 100 (2) K
0.37 × 0.19 × 0.16 mm

#### Data collection

Bruker SMART APEX CCD diffractometer
Absorption correction: multi-scan (*SADABS*; Bruker, 2007 ▶) *T*_min_ = 0.887, *T*_max_ = 0.986
12548 measured reflections
3083 independent reflections
2630 reflections with *I* > 2σ(*I*)
*R*_int_ = 0.026

#### Refinement

*R*[*F* ^2^ > 2σ(*F* ^2^)] = 0.041
*wR*(*F* ^2^) = 0.094
*S* = 1.06
3083 reflections
186 parameters
1 restraint
H atoms treated by a mixture of independent and constrained refinement
Δρ_max_ = 0.20 e Å^−3^
Δρ_min_ = −0.19 e Å^−3^

### 

Data collection: *APEX2* (Bruker, 2007 ▶); cell refinement: *SAINT* (Bruker, 2007 ▶); data reduction: *SAINT*; program(s) used to solve structure: *SHELXTL* (Sheldrick, 2008 ▶); program(s) used to refine structure: *SHELXTL*; molecular graphics: *Mercury* (Macrae *et al.*, 2006 ▶); software used to prepare material for publication: *SHELXTL*.

## Supplementary Material

Crystal structure: contains datablocks global, I. DOI: 10.1107/S1600536808033849/hb2803sup1.cif

Structure factors: contains datablocks I. DOI: 10.1107/S1600536808033849/hb2803Isup2.hkl

Additional supplementary materials:  crystallographic information; 3D view; checkCIF report

## Figures and Tables

**Table 1:** Hydrogen-bond geometry (Å, °)

*D*—H⋯*A*	*D*—H	H⋯*A*	*D*⋯*A*	*D*—H⋯*A*
N1—H1⋯O1^i^	0.878 (12)	1.973 (13)	2.7876 (14)	153.9 (13)
C14—H14*B*⋯*Cg*1^ii^	0.99	2.58	3.4754 (15)	151

Symmetry codes: (i) 
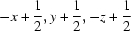
; (ii) 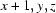. *Cg*1 is the centroid of the C1/C6/C7/C12/N1 ring.

**Fig. 1.**
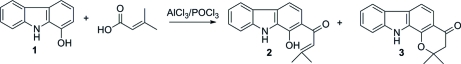
Reaction sequence

**Fig. 2.**
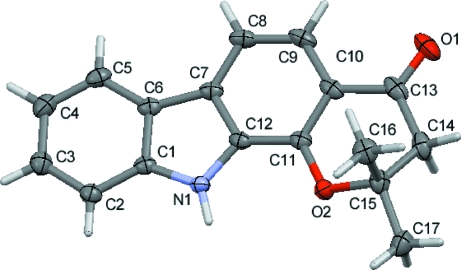
The molecular structure of (I) displaying xx% displacement ellipsoids. H atoms are represented in stick mode.

**Fig. 3.**
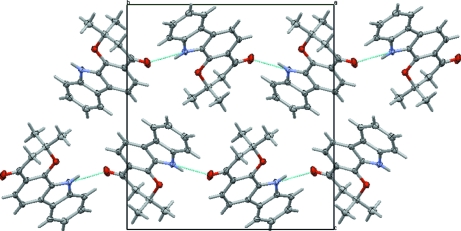
Packing view of (I) down the a axis showing chains built by the N—H···O hydrogen bonds (indicated by blue dashed lines).

## supplementary crystallographic information

### Comment

Pyranocarbazoles such as grinimbine, mupamine, mahanimbine, murrayanol and mahanine have been isolated from plant species of the Rutaceae family (Knölker & Reddy, 2002, and references therein) and these alkaloids possess mosquitocidal, antimicrobial, anti-inflammatory and antioxidant activities (Shanazarov *et al.*, 1989). In general pyranocarbazole alkaloids have a C-13, C-18 or C-23 framework with a C-12 carbazole nucleus as the basic unit in which one carbon is attached as a methyl, formyl, carboxylic or ester group. Another observation is that in many of the pyranocarbazole derivatives isolated so far, the oxygen atom of the pyran ring is attached to carbon atom 2 of the carbazole nucleus to form pyrano[3,2-*a*]carbazoles as in grinimbine. Of the 10 simple carbazole alkaloids isolated so far, five have the oxygen function on carbon 1 (or its equivalent position C 8). The C-18 pyrano[3,2-*a*] alkaloid mupamine posseses a methoxy group at position 8, hence there exists a substrate on which a pyrano[2,3-*a*]carbazole could be built upon in the plant body; however, none of the pyranocarbazoles isolated so far has a pyran ring with oxygen on carbon 1 or its equivalent position C 8.

In this context we aimed to prepare pyrano[2,3-*a*]carbazoles using 1-hydroxycarbazoles as starting synthons under various reaction conditions (Kavitha & Prasad, 2003, and references therein). Using the catalyst mixture AlCl_3_/POCl_3_ along with 1-hydroxycarbazole and 3,3-dimethyacrylic acid as the reactants we were able to generate a mixture of two products *i.e.*, 2-(3,3-dimethylacryloyl)-1-hydroxycarbazole (2) and the title compound 2,2-dimethyl-2,3-dihydropyran*o*-[2,3-*a*]carbazol-4(11*H*)-one (3) (Figure 1).

The single-crystal structure confirmed the formation of the dihydropyran*o*-[2,3-*a*]carbazol-4(11*H*)-one framework as shown in Figure 2. Data collection and structure refinement were unproblematic and all structural parameters (bond lengths, angles, *etc*) are in the expected ranges. The molecules crystallize in a monoclinic setting in *P*2_1_/*n* with four largely planar molecules per unit cell. The plane defined by the *sp*^2^ hybridized carbon atoms, the CH_2_ group and the N and O atoms has an r.m.s. deviation from planarity of only 0.036 Å. Of all the ring C atoms only C15 of the pyran C(Me)_2_ unit is significately out of plane with the atoms of the four fused rings, its deviation being 0.611 (1) Å. The pyran ring thus exhibits a half chair conformation.

One of the methyl groups of the C(Me)_2_ unit is also located close to the average plane of the molecule (C17 with a deviation of 0.264 (1) Å). The other, C16, is however located 2.121 (1) Å away from this plane and thus makes the molecule as a whole not planar and prevents it form forming extensive π-π stacked entities in the solid state. The packing is thus indeed dominated by strong N—H···O hydrogen bonds (Table 1) and a weaker C—H···C (Table 1) interaction.  The unusual C—H···C bond could also be described as a C—H···π interaction [C14—H14*b*···*Cg*1^iii^ = 2.58 Å with *Cg*1 being the centroid of the C1/C6/C7/C12/N1 pyrrole ring and iii = 1 + *x*, *y*, *z*)]. The only noticeable π···π stacking interaction observed is a slipped one between *Cg*3 and *Cg*4^iv^ with a centroid to centroid distance of 3.8425 (8) Å (*Cg*3 and *Cg*4 are C1 to C6 and C7 to C12, respectively, iv = -1 + *x*, *y*, *z*).

The N—H···O hydrogen bonds that dominate the packing of the title compound tie molecules together to infinite chains that extend along the crystallographic *b* axis as shown in Figure 3.

The structures of the 9- and 10-methyl derivatives of (I) are described in Sridharan, Rajendra Prasad & Zeller (2008) and Sridharan, Rajendra Prasad, Ngendahimana *et al.* (2008). For a more detailed comparison of structures and packing of these three compounds, see Sridharan, Rajendra Prasad & Zeller (2008).

### Experimental

1-Hydroxycarbazole (1, 0.001 mol) and 3,3-dimethylacrylicacid (0.001 mol) was dissolved in an ice-cold mixture of AlCl_3_/POCl_3_ (400 mg/ 6 ml) and were kept at room temperature for 24 h. Reaction monitoring by TLC indicated the formation of two compounds. After the completion of reaction (disappearance of starting material), the residue was poured onto ice water. The solid that separated out was filtered, dried and then separated by column chromatography on silica gel using petroleum ether/ ethyl acetate (98:2) as eluants to yield 2-(3,3-dimethylacryloyl)-1-hydroxycarbazole (2) and 2,2-dimethyl-2,3-dihydropyran*o*-[2,3-*a*] carbazol-4(11*H*)-one (3), respectively as yellow prisms. The product 3 thus separated was recrystallized from ethanol (0.106 g, 40%), m.p. 472–474 K.

### Refinement

The amine H atom was located in a difference map and was refined with an N—H distance restraint of 0.88 (2) Å and *U*_iso_ = 1.2 *U*_eq_(N). All other hydrogen atoms were added in calculated positions with C—H bond distances of 0.99 (methylene), 0.95 (aromatic) and 0.98 Å (methyl). They were refined as riding with U_iso_(H) = 1.2U_eq_(C) or 1.5U_eq_(methyl C).

### Crystal data

**Table:**

C_17_H_15_NO_2_	*F*(000) = 560
*M**_r_* = 265.30	*D*_x_ = 1.313 Mg m^−^^3^
Monoclinic, *P*2_1_/*n*	Mo *K*α radiation, λ = 0.71073 Å
Hall symbol: -P 2yn	Cell parameters from 4649 reflections
*a* = 5.9926 (5) Å	θ = 2.6–27.5°
*b* = 14.3368 (12) Å	µ = 0.09 mm^−^^1^
*c* = 15.6839 (13) Å	*T* = 100 K
β = 95.270 (1)°	Plate, yellow
*V* = 1341.78 (19) Å^3^	0.37 × 0.19 × 0.16 mm
*Z* = 4	

### Data collection

**Table:**

Bruker APEX CCD diffractometer	3083 independent reflections
Radiation source: fine-focus sealed tube	2630 reflections with *I* > 2σ(*I*)
graphite	*R*_int_ = 0.026
ω scans	θ_max_ = 27.5°, θ_min_ = 1.9°
Absorption correction: multi-scan (SADABS; Bruker, 2007)	*h* = −7→7
*T*_min_ = 0.887, *T*_max_ = 0.986	*k* = −18→18
12548 measured reflections	*l* = −20→20

### Refinement

**Table:**

Refinement on *F*^2^	Primary atom site location: structure-invariant direct methods
Least-squares matrix: full	Secondary atom site location: difference Fourier map
*R*[*F*^2^ > 2σ(*F*^2^)] = 0.041	Hydrogen site location: difmap and geom
*wR*(*F*^2^) = 0.094	H atoms treated by a mixture of independent and constrained refinement
*S* = 1.06	*w* = 1/[σ^2^(*F*_o_^2^) + (0.035*P*)^2^ + 0.4678*P*] where *P* = (*F*_o_^2^ + 2*F*_c_^2^)/3
3083 reflections	(Δ/σ)_max_ < 0.001
186 parameters	Δρ_max_ = 0.20 e Å^−^^3^
1 restraint	Δρ_min_ = −0.19 e Å^−^^3^

### Special details

**Table:**

Geometry. All e.s.d.'s (except the e.s.d. in the dihedral angle between two l.s. planes) are estimated using the full covariance matrix. The cell e.s.d.'s are taken into account individually in the estimation of e.s.d.'s in distances, angles and torsion angles; correlations between e.s.d.'s in cell parameters are only used when they are defined by crystal symmetry. An approximate (isotropic) treatment of cell e.s.d.'s is used for estimating e.s.d.'s involving l.s. planes.
Refinement. Refinement of *F*^2^ against ALL reflections. The weighted *R*-factor *wR* and goodness of fit *S* are based on *F*^2^, conventional *R*-factors *R* are based on *F*, with *F* set to zero for negative *F*^2^. The threshold expression of *F*^2^ > σ(*F*^2^) is used only for calculating *R*-factors(gt) *etc*. and is not relevant to the choice of reflections for refinement. *R*-factors based on *F*^2^ are statistically about twice as large as those based on *F*, and *R*- factors based on ALL data will be even larger.

### Fractional atomic coordinates and isotropic or equivalent isotropic displacement parameters (Å^2^)

**Table:**

	*x*	*y*	*z*	*U*_iso_*/*U*_eq_	
C1	−0.2845 (2)	0.22408 (8)	0.34560 (7)	0.0214 (3)	
C2	−0.4447 (2)	0.29342 (9)	0.35331 (8)	0.0244 (3)	
H2	−0.4468	0.3487	0.3198	0.029*	
C3	−0.6002 (2)	0.27850 (9)	0.41161 (8)	0.0286 (3)	
H3	−0.7127	0.3241	0.4176	0.034*	
C4	−0.5963 (2)	0.19767 (10)	0.46224 (8)	0.0300 (3)	
H4	−0.7037	0.1901	0.5027	0.036*	
C5	−0.4385 (2)	0.12915 (9)	0.45400 (8)	0.0269 (3)	
H5	−0.4370	0.0745	0.4883	0.032*	
C6	−0.2805 (2)	0.14115 (8)	0.39445 (8)	0.0228 (3)	
C7	−0.1010 (2)	0.08444 (8)	0.36710 (7)	0.0221 (3)	
C8	−0.0205 (2)	−0.00614 (9)	0.38728 (8)	0.0266 (3)	
H8	−0.0852	−0.0427	0.4291	0.032*	
C9	0.1525 (2)	−0.04017 (8)	0.34536 (8)	0.0278 (3)	
H9	0.2088	−0.1008	0.3590	0.033*	
C10	0.2501 (2)	0.01264 (8)	0.28208 (8)	0.0241 (3)	
C11	0.1683 (2)	0.10152 (8)	0.25993 (7)	0.0209 (3)	
C12	−0.0055 (2)	0.13669 (8)	0.30376 (7)	0.0205 (2)	
C13	0.4420 (2)	−0.02233 (9)	0.24051 (9)	0.0287 (3)	
C14	0.5333 (2)	0.04206 (10)	0.17638 (9)	0.0299 (3)	
H14A	0.5983	0.0041	0.1321	0.036*	
H14B	0.6559	0.0796	0.2058	0.036*	
C15	0.3590 (2)	0.10787 (9)	0.13255 (8)	0.0259 (3)	
C16	0.1830 (2)	0.05565 (10)	0.07445 (8)	0.0303 (3)	
H16A	0.1108	0.0086	0.1079	0.045*	
H16B	0.2552	0.0250	0.0283	0.045*	
H16C	0.0701	0.0998	0.0498	0.045*	
C17	0.4667 (2)	0.18495 (10)	0.08453 (9)	0.0338 (3)	
H17A	0.3501	0.2262	0.0578	0.051*	
H17B	0.5513	0.1576	0.0401	0.051*	
H17C	0.5685	0.2209	0.1245	0.051*	
N1	−0.11817 (17)	0.21994 (7)	0.29049 (6)	0.0208 (2)	
H1	−0.073 (2)	0.2679 (9)	0.2619 (9)	0.025*	
O1	0.53069 (18)	−0.09781 (7)	0.25866 (7)	0.0406 (3)	
O2	0.24499 (14)	0.15638 (6)	0.19818 (5)	0.0237 (2)	

### Atomic displacement parameters (Å^2^)

**Table:**

	*U*^11^	*U*^22^	*U*^33^	*U*^12^	*U*^13^	*U*^23^
C1	0.0231 (6)	0.0209 (6)	0.0197 (6)	−0.0045 (5)	−0.0007 (5)	−0.0018 (5)
C2	0.0267 (6)	0.0219 (6)	0.0243 (6)	−0.0012 (5)	0.0006 (5)	−0.0017 (5)
C3	0.0275 (7)	0.0294 (7)	0.0289 (7)	−0.0016 (5)	0.0028 (5)	−0.0065 (5)
C4	0.0294 (7)	0.0357 (7)	0.0258 (6)	−0.0094 (6)	0.0066 (5)	−0.0050 (6)
C5	0.0329 (7)	0.0261 (6)	0.0217 (6)	−0.0099 (5)	0.0021 (5)	−0.0004 (5)
C6	0.0264 (6)	0.0212 (6)	0.0200 (6)	−0.0058 (5)	−0.0023 (5)	−0.0015 (5)
C7	0.0268 (6)	0.0193 (6)	0.0192 (6)	−0.0045 (5)	−0.0032 (5)	−0.0002 (4)
C8	0.0372 (7)	0.0196 (6)	0.0216 (6)	−0.0049 (5)	−0.0042 (5)	0.0026 (5)
C9	0.0387 (7)	0.0159 (6)	0.0265 (6)	0.0017 (5)	−0.0096 (6)	0.0000 (5)
C10	0.0279 (6)	0.0185 (6)	0.0241 (6)	0.0011 (5)	−0.0075 (5)	−0.0046 (5)
C11	0.0227 (6)	0.0188 (6)	0.0202 (6)	−0.0029 (5)	−0.0035 (5)	−0.0019 (4)
C12	0.0236 (6)	0.0164 (5)	0.0205 (6)	−0.0021 (4)	−0.0031 (5)	−0.0006 (4)
C13	0.0293 (7)	0.0236 (6)	0.0308 (7)	0.0047 (5)	−0.0096 (5)	−0.0109 (5)
C14	0.0236 (6)	0.0352 (7)	0.0302 (7)	0.0051 (5)	−0.0017 (5)	−0.0112 (6)
C15	0.0245 (6)	0.0292 (7)	0.0239 (6)	0.0003 (5)	0.0018 (5)	−0.0069 (5)
C16	0.0306 (7)	0.0344 (7)	0.0247 (6)	−0.0013 (6)	−0.0041 (5)	−0.0047 (5)
C17	0.0303 (7)	0.0396 (8)	0.0327 (7)	−0.0031 (6)	0.0091 (6)	−0.0039 (6)
N1	0.0240 (5)	0.0160 (5)	0.0226 (5)	−0.0007 (4)	0.0032 (4)	0.0024 (4)
O1	0.0426 (6)	0.0243 (5)	0.0530 (7)	0.0112 (4)	−0.0062 (5)	−0.0103 (5)
O2	0.0258 (4)	0.0218 (4)	0.0239 (4)	−0.0001 (3)	0.0048 (4)	−0.0019 (3)

### Geometric parameters (Å, °)

**Table:**

C1—N1	1.3790 (16)	C10—C13	1.4617 (18)
C1—C2	1.3952 (17)	C11—O2	1.3599 (14)
C1—C6	1.4135 (17)	C11—C12	1.3942 (17)
C2—C3	1.3808 (18)	C12—N1	1.3775 (15)
C2—H2	0.9500	C13—O1	1.2275 (16)
C3—C4	1.4039 (19)	C13—C14	1.505 (2)
C3—H3	0.9500	C14—C15	1.5239 (18)
C4—C5	1.378 (2)	C14—H14A	0.9900
C4—H4	0.9500	C14—H14B	0.9900
C5—C6	1.4005 (18)	C15—O2	1.4625 (15)
C5—H5	0.9500	C15—C17	1.5148 (19)
C6—C7	1.4444 (18)	C15—C16	1.5252 (17)
C7—C12	1.4071 (17)	C16—H16A	0.9800
C7—C8	1.4111 (17)	C16—H16B	0.9800
C8—C9	1.3679 (19)	C16—H16C	0.9800
C8—H8	0.9500	C17—H17A	0.9800
C9—C10	1.4164 (19)	C17—H17B	0.9800
C9—H9	0.9500	C17—H17C	0.9800
C10—C11	1.3976 (17)	N1—H1	0.878 (12)
			
N1—C1—C2	128.89 (11)	N1—C12—C7	110.03 (11)
N1—C1—C6	109.04 (11)	C11—C12—C7	121.75 (11)
C2—C1—C6	121.99 (12)	O1—C13—C10	122.73 (13)
C3—C2—C1	117.32 (12)	O1—C13—C14	121.29 (13)
C3—C2—H2	121.3	C10—C13—C14	115.92 (11)
C1—C2—H2	121.3	C13—C14—C15	113.88 (11)
C2—C3—C4	121.68 (13)	C13—C14—H14A	108.8
C2—C3—H3	119.2	C15—C14—H14A	108.8
C4—C3—H3	119.2	C13—C14—H14B	108.8
C5—C4—C3	120.76 (12)	C15—C14—H14B	108.8
C5—C4—H4	119.6	H14A—C14—H14B	107.7
C3—C4—H4	119.6	O2—C15—C17	104.54 (10)
C4—C5—C6	119.14 (12)	O2—C15—C14	108.80 (10)
C4—C5—H5	120.4	C17—C15—C14	111.72 (11)
C6—C5—H5	120.4	O2—C15—C16	108.18 (10)
C5—C6—C1	119.08 (12)	C17—C15—C16	111.33 (11)
C5—C6—C7	134.14 (12)	C14—C15—C16	111.92 (11)
C1—C6—C7	106.77 (11)	C15—C16—H16A	109.5
C12—C7—C8	119.70 (12)	C15—C16—H16B	109.5
C12—C7—C6	105.79 (10)	H16A—C16—H16B	109.5
C8—C7—C6	134.42 (12)	C15—C16—H16C	109.5
C9—C8—C7	118.63 (12)	H16A—C16—H16C	109.5
C9—C8—H8	120.7	H16B—C16—H16C	109.5
C7—C8—H8	120.7	C15—C17—H17A	109.5
C8—C9—C10	121.73 (11)	C15—C17—H17B	109.5
C8—C9—H9	119.1	H17A—C17—H17B	109.5
C10—C9—H9	119.1	C15—C17—H17C	109.5
C11—C10—C9	120.25 (12)	H17A—C17—H17C	109.5
C11—C10—C13	118.29 (12)	H17B—C17—H17C	109.5
C9—C10—C13	121.42 (11)	C12—N1—C1	108.36 (10)
O2—C11—C12	117.29 (10)	C12—N1—H1	125.7 (9)
O2—C11—C10	124.80 (11)	C1—N1—H1	124.3 (9)
C12—C11—C10	117.90 (11)	C11—O2—C15	115.87 (10)
N1—C12—C11	128.09 (11)		
			
N1—C1—C2—C3	177.14 (12)	C10—C11—C12—N1	−176.92 (11)
C6—C1—C2—C3	0.56 (18)	O2—C11—C12—C7	178.19 (10)
C1—C2—C3—C4	0.94 (19)	C10—C11—C12—C7	−1.42 (17)
C2—C3—C4—C5	−1.4 (2)	C8—C7—C12—N1	176.09 (10)
C3—C4—C5—C6	0.24 (19)	C6—C7—C12—N1	−1.00 (13)
C4—C5—C6—C1	1.21 (18)	C8—C7—C12—C11	−0.15 (18)
C4—C5—C6—C7	−177.49 (13)	C6—C7—C12—C11	−177.24 (10)
N1—C1—C6—C5	−178.83 (11)	C11—C10—C13—O1	176.81 (12)
C2—C1—C6—C5	−1.65 (17)	C9—C10—C13—O1	−1.12 (19)
N1—C1—C6—C7	0.20 (13)	C11—C10—C13—C14	−0.30 (16)
C2—C1—C6—C7	177.38 (11)	C9—C10—C13—C14	−178.23 (11)
C5—C6—C7—C12	179.29 (13)	O1—C13—C14—C15	154.37 (12)
C1—C6—C7—C12	0.48 (13)	C10—C13—C14—C15	−28.47 (15)
C5—C6—C7—C8	2.8 (2)	C13—C14—C15—O2	52.15 (14)
C1—C6—C7—C8	−175.98 (13)	C13—C14—C15—C17	167.05 (11)
C12—C7—C8—C9	1.22 (17)	C13—C14—C15—C16	−67.34 (14)
C6—C7—C8—C9	177.30 (13)	C11—C12—N1—C1	177.08 (11)
C7—C8—C9—C10	−0.71 (18)	C7—C12—N1—C1	1.15 (13)
C8—C9—C10—C11	−0.88 (18)	C2—C1—N1—C12	−177.75 (12)
C8—C9—C10—C13	177.01 (11)	C6—C1—N1—C12	−0.82 (13)
C9—C10—C11—O2	−177.66 (11)	C12—C11—O2—C15	−157.25 (10)
C13—C10—C11—O2	4.38 (17)	C10—C11—O2—C15	22.33 (16)
C9—C10—C11—C12	1.92 (17)	C17—C15—O2—C11	−168.60 (10)
C13—C10—C11—C12	−176.04 (10)	C14—C15—O2—C11	−49.13 (13)
O2—C11—C12—N1	2.69 (18)	C16—C15—O2—C11	72.67 (13)

### Hydrogen-bond geometry (Å, °)

**Table:**

*D*—H···*A*	*D*—H	H···*A*	*D*···*A*	*D*—H···*A*
N1—H1···O1^i^	0.88 (1)	1.97 (1)	2.7876 (14)	154 (1)
C14—H14B···Cg1^ii^	0.99	2.58	3.4754 (15)	151

Symmetry codes: (i) −*x*+1/2, *y*+1/2, −*z*+1/2; (ii) *x*+1, *y*, *z*.
